# Supplementary material for: A Quaternary mixed oxide protective scaffold for ruthenium during oxygen evolution reaction in acidic media
Source: Commun Eng. 2023 May 22;2:28. doi: 10.1038/s44172-023-00080-5 (PMC10955812; doi:10.1038/s44172-023-00080-5)
Supplement: Supplementary file 2 — Supplementary information file [file 44172_2023_80_MOESM2_ESM.pdf]

Supplementary Information

A Quaternary Mixed Oxide Protective Scaffold for Ruthenium During Oxygen Evolution Reaction in Acidic Media

Alexis Piñeiro-García,[1] Xiuyu Wu,[1] Mouna Rafei,[1] Paul Jonathan Mörk,[1,2] and Eduardo Gracia-Espino.[1,*]

[1] *Department of Physics, Umeå University, SE-901 87 Umeå, Sweden.*

[2] *Faculty of physics and astronomy, Julius-Maximilians-Universität Würzburg, Germany*

[*]Corresponding author: Eduardo Gracia-Espino (eduardo.gracia@umu.se)

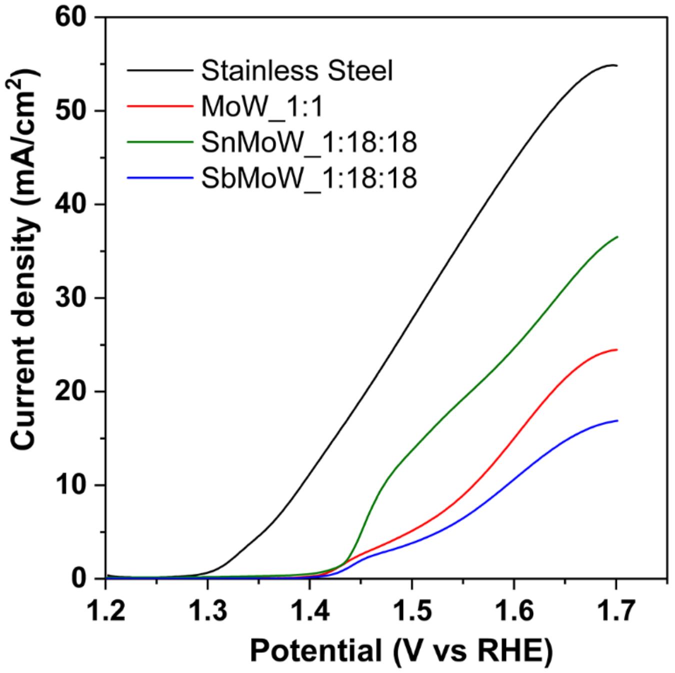

Figure S1. Linear sweep voltammetry (LSV) for the coatings MoW, SnMoW and SbMoW (atomic ratio showed in the Figure) as well as stainless steel at pH=0. *The SS exhibit a significant current density at 1.3V vs RHE due to transpasivation reaction under acidic conditions. In principle, the protective coating should retard the transpasivation of SS and reduce the current density. As can be seen, the three combination of metal oxides tested led to an increase in the onset (120 mV) meanwhile the current density was reduced. These results point that a reduction of the transspasivation of SS was achieved due to the protective environment created by the metal oxides deposited on it.*

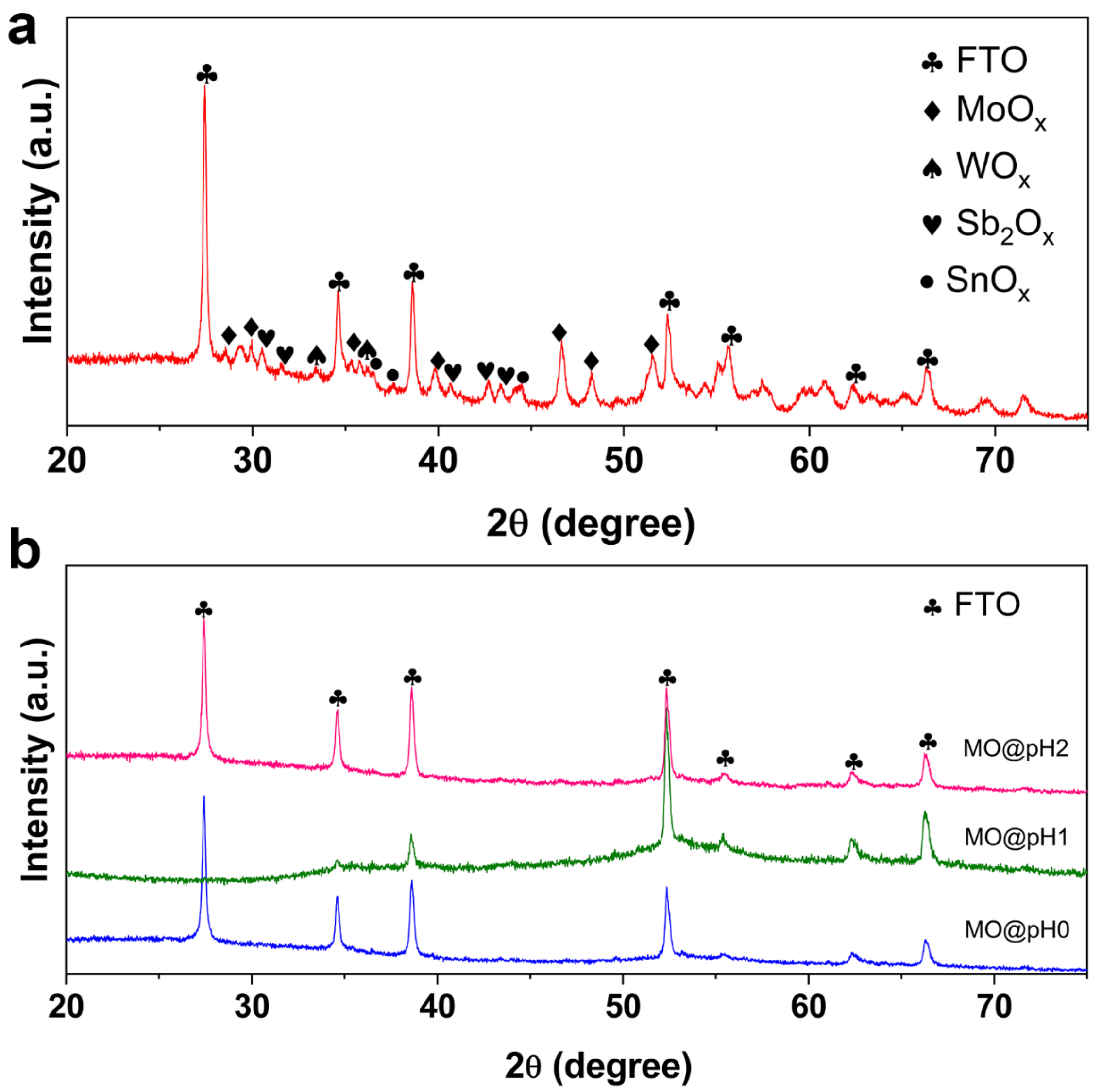

Figure S2. X-ray powder diffraction of MO before and after the stress test. *(a) Pristine coating deposited onto FTO. (b) MO coating after the stress test at different pH conditions.*

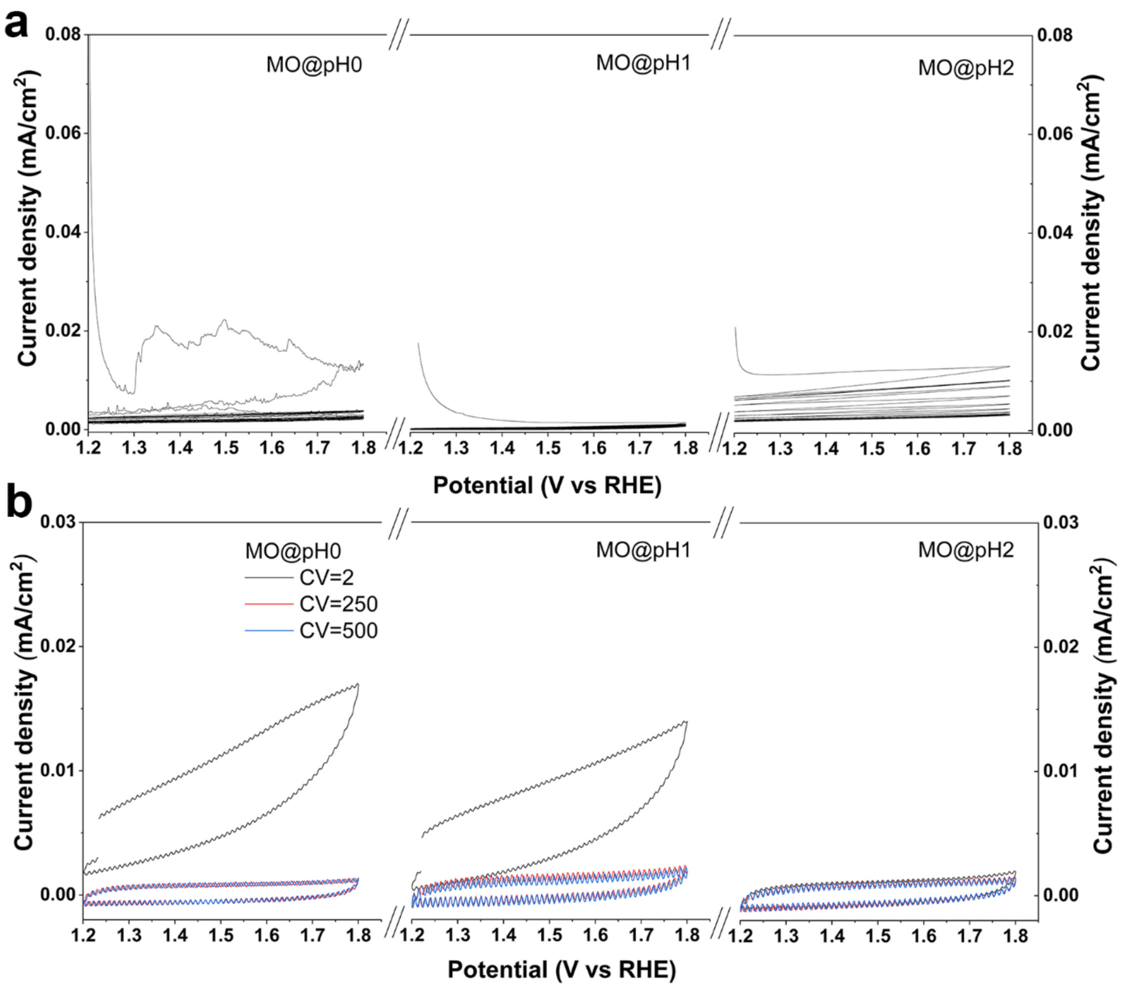

***Figure S3. Stability test of MO at different pH and scan rates.** (a) Cyclic voltammograms (CVs) of MO deposited on FTO substrates evaluated at different pH conditions. (b) 500 CVs measured at 100 mV s^{-1} scan rate, only CV 2, 250 and 500 are shown.*

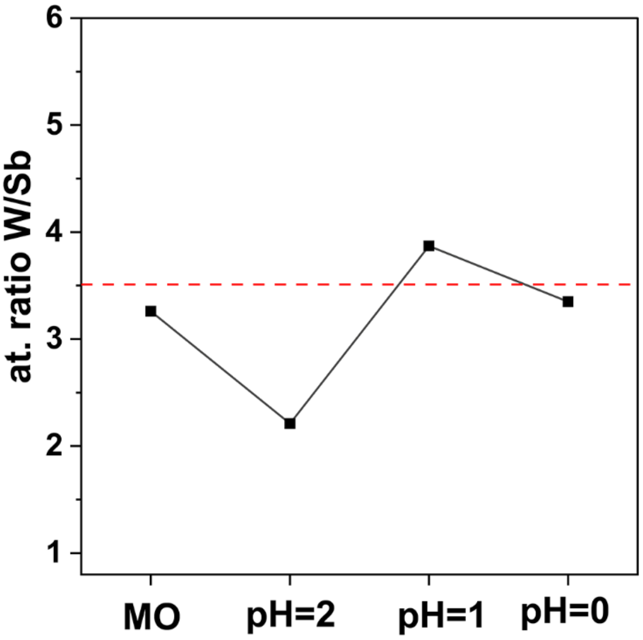

Figure S4. Variation of W/Sb atomic ratio of MO as produced and after stress test at various pH. *W/Sb atomic ratio calculated by EDX. The red-dot line represents the molar ratio in the precursor solution.*

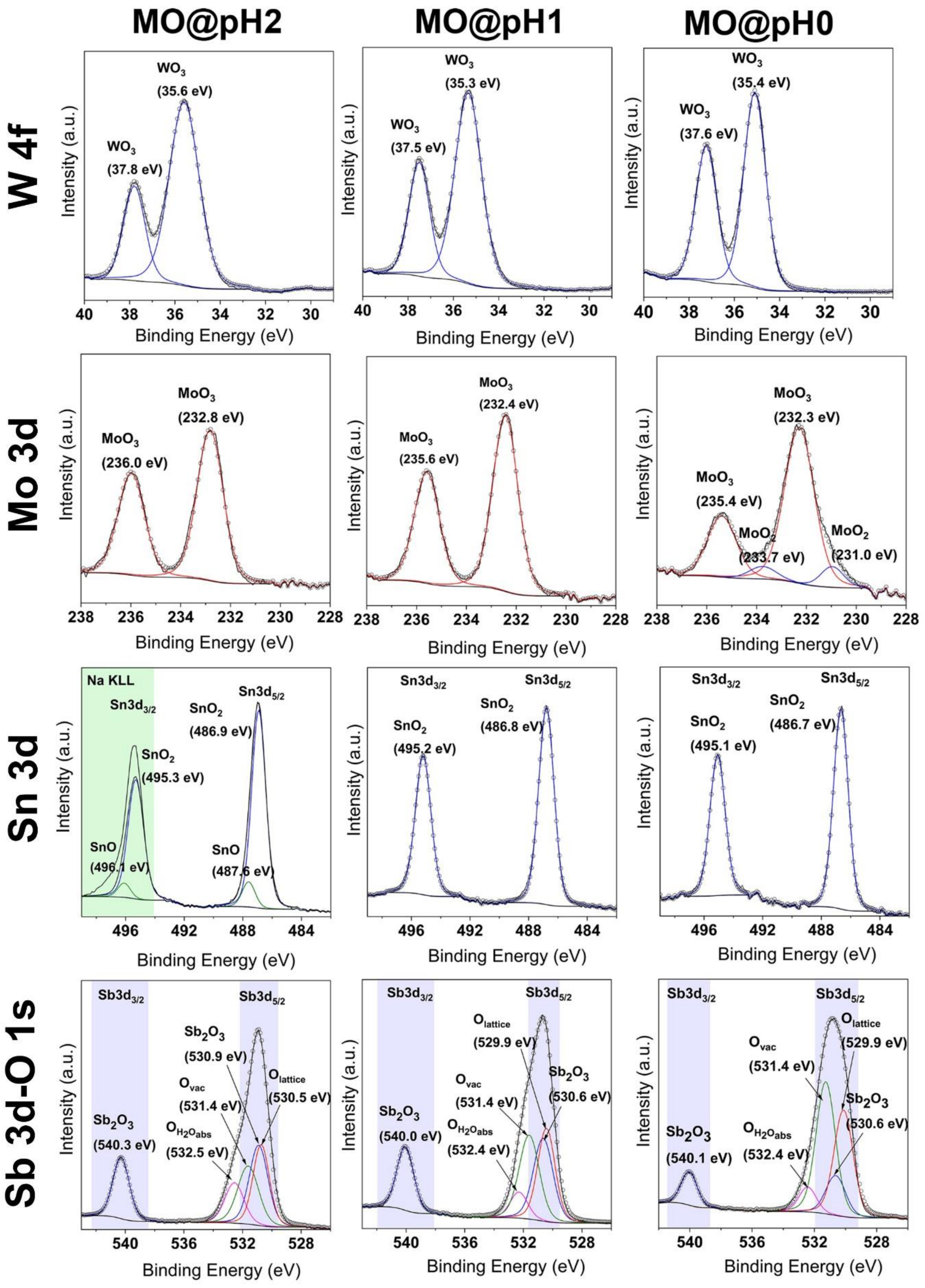

Figure S5. XPS studies of MO after the stress test at various pH. *High resolution XPS core-level spectra of Sb 3d/O 1s, Sn 3d, Mo 3d and W 4f of MO@pH0, MO@pH1 and MO@pH2 after 500 CVs.*

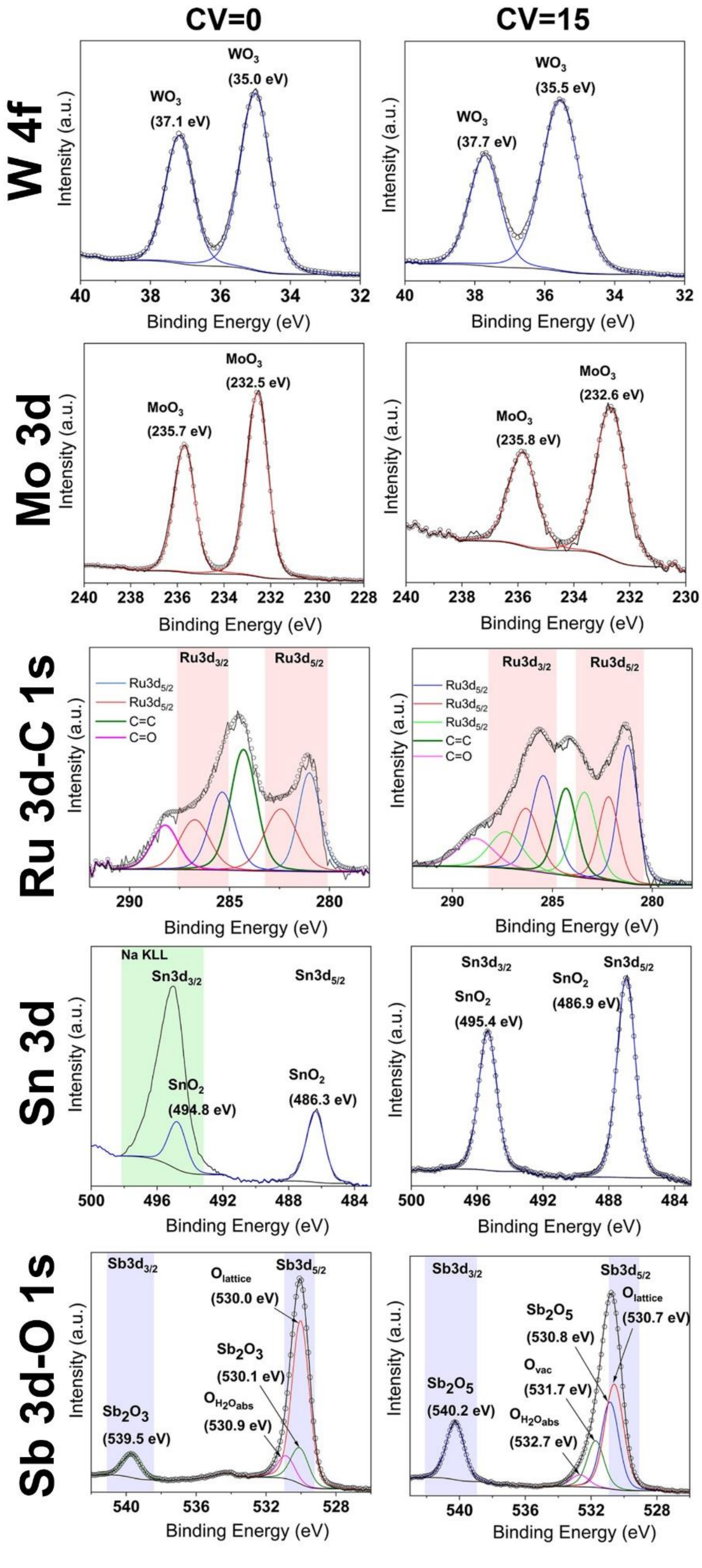

Figure S6. XPS studies of Ru-Mo@24 before and after the CV test. *High resolution XPS core-level spectra of Sb 3d/O 1s, Sn 3d, Mo 3d and W 4f before and after electrochemical measurements at pH=0.*

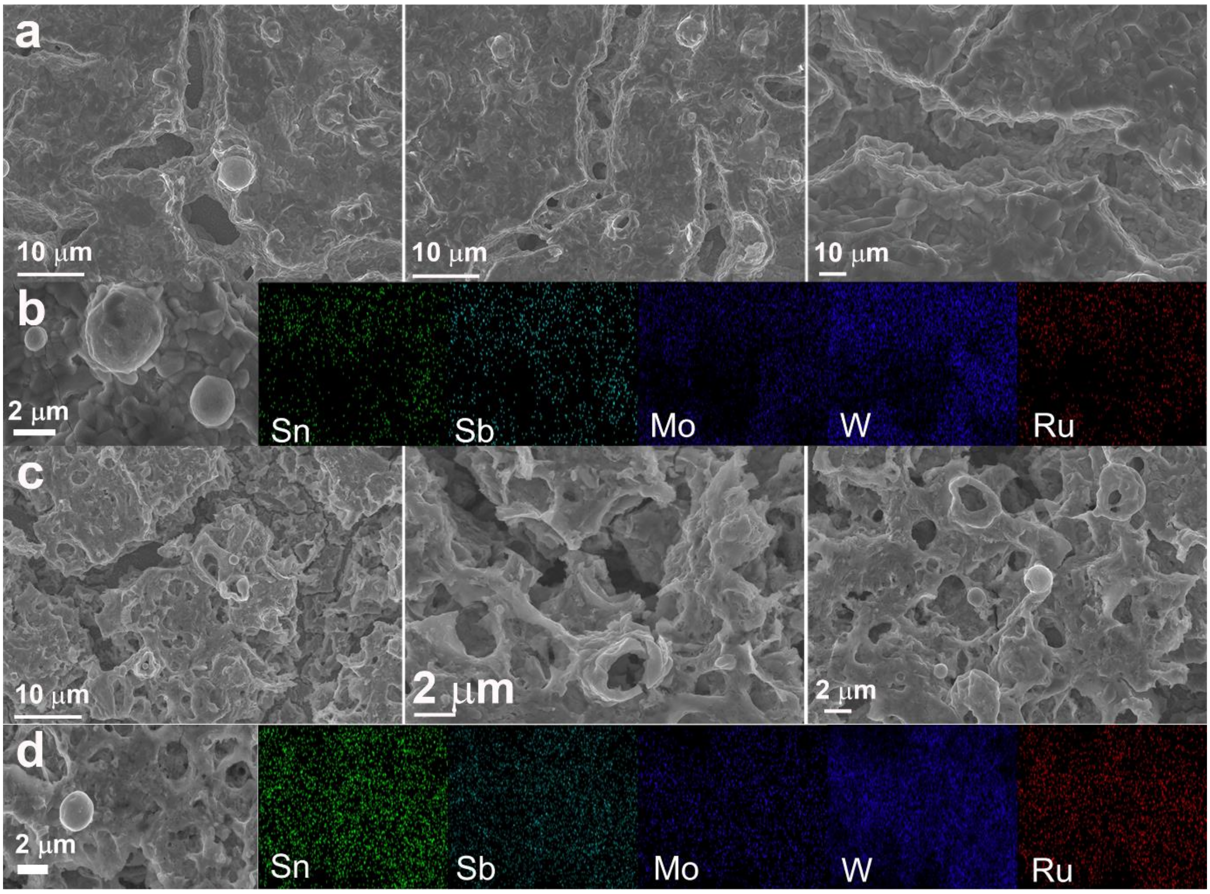

Figure S7. **SEM studies of Ru-MO@24h before and after the electrochemical stress test.** *(a-b) Ru-MO@24h before OER. (c-d) Ru-MO@24h after 15 CV scans.*

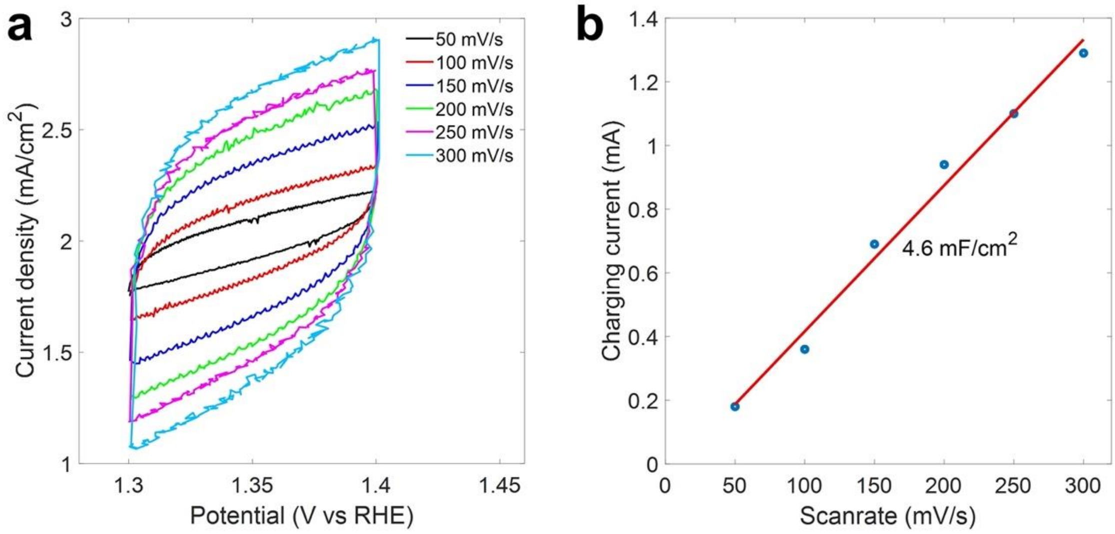

Figure S8. Determination of the electrochemical surface area of Ru-Mo@Ti. *(a) CVs measurements in the non-faradaic region (1.3-1.4V vs RHE) at different scan rates and (b) linear fitting of the capacitive currents vs scan rates (geometric area of the electrode is equal to 1 cm^2).*

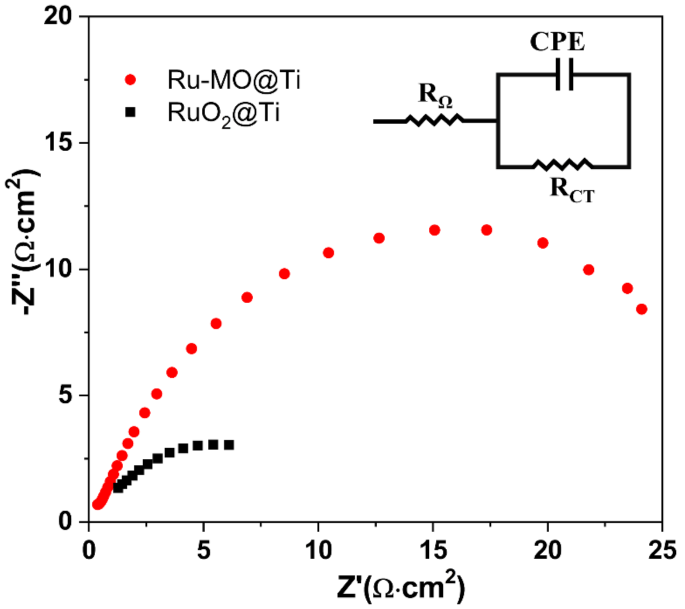

Figure S9. Electrochemical impedance spectroscopy of RuO_2@Ti and Ru-MO@Ti. *Nyquist plot evaluated at 1.7 V vs RHE; the inset shows the equivalent circuit model.*

Table S1. Comparison of the ohmic resistance (R_Ω) and charge transfer resistance (R_{CT}) for *RuO_2@Ti* and *Ru-MO@Ti* evaluated at 1.7V vs RHE.

**Sample**	**R_Ω (Ω cm^2)**	**R_{CT} (Ω cm^2)**
RuO_2@Ti	0.6	8.5
Ru-MO@Ti	0.25	32.8

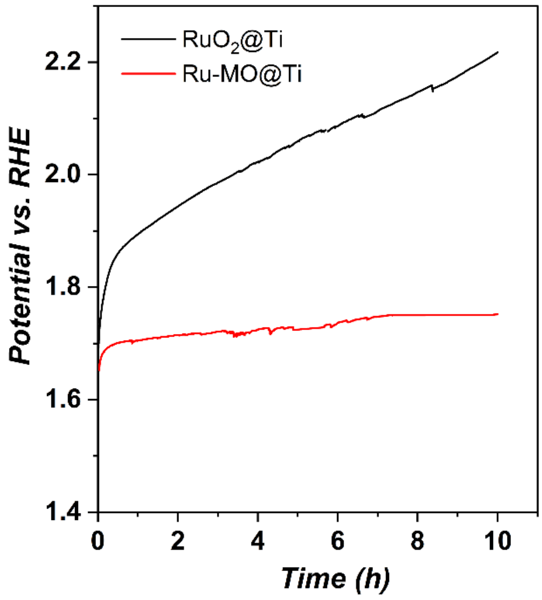

Figure S10. Stability test of Ru-MO@Ti and RuO_2@Ti by a chronopotentiometry study. *Applied current of 2.5 mA cm^{-2} at pH=0.*

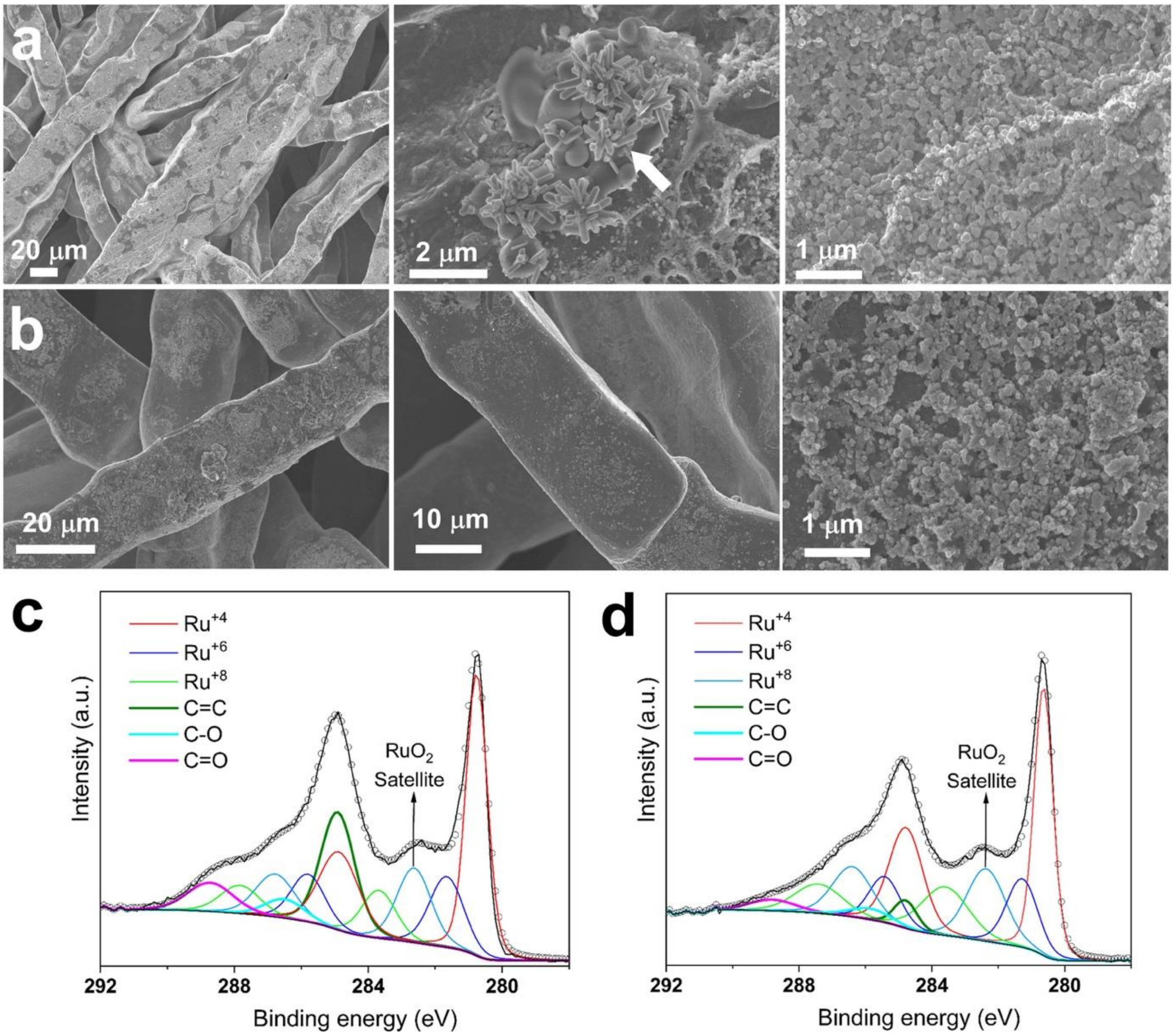

Figure S11. SEM and XPS studies of RuO_2@Ti. *(a) SEM images before the stability test. (b) SEM images after the stability test. High resolution XPS core-level spectra of Ru 3d for RuO_2@Ti (c) before and (d) after the stability test.*
